# Motivation among Teenage Football Players: A Longitudinal Investigation throughout a Competitive Season

**DOI:** 10.3390/ejihpe13090124

**Published:** 2023-09-04

**Authors:** Filipe Rodrigues, Diogo Monteiro, Rui Matos, Miguel Jacinto, Raúl Antunes, Nuno Amaro

**Affiliations:** 1 ESECS—Polytechnic of Leiria, 2411-901 Leiria, Portugal; diogo.monteiro@ipleiria.pt (D.M.); rui.matos@ipleiria.pt (R.M.); miguel.s.jacinto@ipleiria.pt (M.J.); raul.antunes@ipleiria.pt (R.A.); nuno.amaro@ipleiria.pt (N.A.); 2 Life Quality Research Centre (CIEQV), 2400-901 Leiria, Portugal; 3 Research Center in Sport, Health, and Human Development (CIDESD), 5000-558 Vila Real, Portugal; 4 Center for Innovative Care and Health Technology (ciTechCare), 2415-396 Leiria, Portugal

**Keywords:** self-determination theory, motivation, competitive season, football, teenage

## Abstract

The present study aimed to investigate the dynamic changes in behavioral regulations among teenage football players throughout a competitive season, with three measurement points (T1, T2, and T3). The total sample size for the study comprised 108 participants (78 male; 30 female). The participants’ mean age was 14.31 (SD = 1.48). A MANOVA repeated measures analysis was performed within the group for all six behavioral regulations based on self-determination theory. The results of the study revealed noteworthy patterns in the behavioral regulations and intrinsic motivation scores over the competitive season. Identified regulation demonstrated a significant increase from T1 to T3 (*p* < 0.01, η^2p^ = 0.13). Intrinsic motivation displayed a significant decrease over the same period from T1 to T3 (*p* < 0.01, η^2p^ = 0.12). On the other hand, amotivation, external regulation, introjected regulation, and integrated regulation did not show significant changes between the three time points. The findings have practical implications for coaches and sports practitioners, as they highlight the need to create a supportive and autonomy-promoting environment to sustain athletes’ intrinsic motivation throughout a competitive season. Also, recognizing the increase in identified regulation can inform strategies that align team goals and training sessions with players’ individual values, thereby enhancing their commitment and dedication to the team’s success.

## 1. Introduction

Football, recognized as one of the world’s most beloved sports, possesses an enchanting appeal that captivates players and fans on a global scale. The sport’s dynamic gameplay, impressive skills, and unwavering competitive spirit converge to create an exhilarating spectacle [1]. However, beyond the superficial excitement, there exists a deeper recognition of the significance of motivation, particularly in the context of teenage football during a competitive season. Motivation assumes a pivotal role in driving the behavior and experiences of young football players throughout the competitive season [2,3,4]. It serves as the underlying impetus that propels individuals to engage, perform, and persevere in the face of challenges. Gaining an understanding of the diverse forms of motivation that influence young athletes can yield invaluable insights into their overall engagement, development, and enjoyment of the sport [5,6].

### 1.1. Motivation Based on Self-Determination Theory

Motivation exhibits a dynamic and evolving nature, following a continuum over time. In various pursuits, including sports like football, individuals’ motivation undergoes shifts that influence their level of engagement and dedication during different stages. To comprehensively understand motivation, the self-determination theory [7] provides a robust framework. Within this framework, motivation can manifest in six distinct ways, ranging from more self-determined to less self-determined forms, broadly categorized as intrinsic motivation, extrinsic motivation, and amotivation [7,8,9].

At the highest level of self-determination, intrinsic motivation drives individuals to engage in behavior purely for the pleasure, fun, and sense of discovery it brings, reflecting an expression of their true selves and identity. For instance, a young football player who is intrinsically motivated finds immense joy and satisfaction in the game itself. Moving along the continuum, various forms of extrinsic motivation emerge, where behavior is influenced by external rewards or pressures. Among these, integrated regulation represents the most self-determined form, where individuals willingly engage in behavior aligned with their beliefs and needs. For example, a teenage football player values the rewards that come from playing soccer, such as the thrill of winning and enhancing physical performance. Next, identified regulation involves recognizing the significance and benefits of a behavior, even if the individual may not inherently enjoy it. They consciously accept the conduct and display a relative willingness to act. For instance, a young football player acknowledges the importance of physical fitness and team spirit, recognizing how participating in football contributes to those goals. Introjected regulation arises from internal forces, such as guilt and fear, and is less self-determined. Individuals may engage in behavior out of a sense of obligation or to avoid negative feelings like shame. For instance, a teenage football player might feel a sense of guilt or fear of disappointing their parents, coach, or teammates if they do not perform well on the field. Further along the continuum, external regulation represents the least self-determined form of motivation, where individuals perform behaviors primarily to meet external needs or obtain rewards, such as prizes or monetary resources. For example, a young football player might be motivated to play for external rewards like winning trophies, earning medals, or receiving praise from others. At the extreme end of the continuum, amotivation emerges, where individuals lack the regulation or intention to engage in a specific behavior. They may perform the behavior without proactive thinking or intention, feeling a sense of ineptitude and a lack of control. For example, a teenage football player who has lost interest and no longer feels motivated to participate in football.

### 1.2. Behavioral Regulation: A Continuum of Influence over Time

During a competitive season in teenage football, the motivations elucidated by the self-determination theory exhibit dynamic characteristics and a susceptibility to changes influenced by various factors. The competitive nature of the season itself significantly shapes the motivational experiences of athletes [10,11,12,13]. Throughout the season, players encounter abundant opportunities for intrinsic motivation to flourish as they derive joy from engaging in matches and witnessing their progress. Positive interactions with coaches as well as the process of overcoming challenges further enhance their intrinsic motivation. However, when facing tough opponents, injuries, or setbacks, there may be a temporary dampening of their intrinsic motivation. Integrated regulation gains strength as players become more invested in the team’s success and recognize the sport’s role in their personal growth. Cohesive teamwork, supportive coaching, and a positive team atmosphere contribute to players embracing football as an integral part of their lives. Nonetheless, external pressures or conflicts, such as academic demands or injuries, may lead to fluctuations in integrated regulation. Similarly, identified regulation is reinforced as players deepen their understanding of the benefits associated with their participation, such as improved performance and enhanced social connections. Supportive feedback from coaches and parents also contributes to a positive shift in identified regulation. However, conflicting priorities or a sense of disconnection from the sport’s relevance may influence this form of motivation. Within the intense atmosphere of the competitive season, players may experience internal pressure with introjected regulation. Supportive coaching styles can mitigate these feelings, while critical or punitive approaches could exacerbate them. External regulation is influenced by external rewards and incentives, such as winning matches or receiving praise. The significance placed on these outcomes and the competitiveness of matches can lead to fluctuations in external regulation. Throughout the season, players may also encounter amotivation, a lack of motivation to engage in football due to challenges like high-pressure matches and demanding training schedules [13,14]. Supportive coaching, autonomy in decision making, and opportunities for skill mastery can mitigate amotivation, while a lack of support or excessive pressure may perpetuate it. It is essential to acknowledge that the discussion of changes in motivations over time within the context of teenage football during a competitive season is predominantly theoretical [7,8]. While existing research offers valuable insights, empirical evidence regarding the dynamics of motivations throughout the competitive season is limited [6,11,15]. Future longitudinal studies are warranted to provide a more comprehensive understanding of how motivations fluctuate across different stages of engagement in teenage football.

### 1.3. Current Study

To the best of our knowledge, no study has yet examined the fluctuations in the motivations of teenage football players over a competitive season. Most existing research on the key motivational determinants in teenage football has utilized a cross-sectional design, providing insights into associations with other psychological and physiological aspects of football but not capturing the dynamic nature of motivations over time [4,6,15,16,17]. In contrast, longitudinal studies may offer the potential to unravel how motivations, including all regulations, evolve as players progress through different stages of their football careers [18,19,20,21]. In addition to the limitations outlined above, it is worth noting that while some studies have examined motivational fluctuations in team sports, many of these investigations have been conducted in sports other than teenage football [19,22]. Furthermore, although several studies have explored specific aspects of motivational determinants in teenage football, none have comprehensively assessed all six behavioral regulations across three measurements throughout a competitive season [19]. Understanding the trajectories of motivations throughout the season can be crucial in developing strategies to promote sustained engagement and well-being in football.

The objective of this study is to conduct a longitudinal investigation aimed at exploring the dynamic nature of motivations, including all six regulations, in teenage football players throughout the duration of a competitive season. By observing how intrinsic motivation, integrated regulation, identified regulation, introjected regulation, external regulation, and amotivation may change over time, evidence-based interventions, training programs, and policies can be crafted to maximize the positive impact of football.

Theoretically, during the competitive season for teenage football players, it is expected that their motivations will undergo changes and fluctuations. As players progress through the season, several hypotheses can be proposed based on theoretical considerations. It is hypothesized that intrinsic motivation will increase during the competitive season. As players engage in regular training, competition, and team bonding experiences, their passion for the sport may strengthen, leading to higher levels of intrinsic motivation. It is hypothesized that integrated regulation will increase steadily during the competitive season. As players align their football involvement with their personal values and identity, integrated regulation is expected to strengthen over time. It is hypothesized that identified regulation will also increase throughout the season. It is hypothesized that introjected regulation may initially increase but could stabilize or decrease over the season. As players face challenges or receive external feedback, they may experience varying levels of self-imposed pressure, which might influence introjected regulation. It is hypothesized that external regulation may have mixed patterns. At the beginning of the season, external factors like praise, recognition, or competition might influence motivation. It is hypothesized that amotivation may decrease throughout the season. As players experience the benefits of regular engagement and teamwork, their indifference towards football may reduce. These hypotheses are based on the assumption that engagement in teenage sport and the associated experiences during the competitive season will contribute to changes in the players’ motivations [11,13,15,22].

## 2. Methods

### 2.1. Sample Size Calculations

The a priori sample size calculations were employed to determine the minimum required sample size in a rigorous manner. Given the use of G*Power 3.1 [23] and the conducted a priori power analysis, the test family chosen was “F tests,” with the statistical test being “MANOVA: repeated measures.” We considered factors (number of measurements) of 3, and the effect size (f) was set at 0.25. Additionally, we chose an alpha error probability of 0.05 and a desired statistical power (1-beta error probability) of 0.95, with a single group and three measurements. The software indicated that a total sample size of 86 participants would result in an actual statistical power of 0.95. This means that with 86 participants, there is a high probability (95%) of detecting a significant effect if it truly exists in the population. Considering data limitations, we collected an additional 10% of data beyond the estimated minimum sample size to ensure adequate statistical power and account for any potential challenges during data collection.

### 2.2. Data Collection Procedures

Using a convenience sampling approach, we established contact with a local football club and sought permission from the director to conduct our study. The research was conducted in collaboration with a regional football academy that caters to both male and female students. This academy, while not paralleling the intensity of elite professional teenage academies, upholds a structured training regimen, ensuring consistent development and growth for its attendees. Upon receiving a positive response, we proceeded to communicate with the parents or legal guardians of the participating adolescents. We provided them with a comprehensive presentation about the study’s nature, ethical considerations, and data collection procedures. Informed consent was obtained from the parents or legal guardians, granting permission for their children’s inclusion in the research. Before distributing the questionnaires, we explained the study’s objectives to all athletes and emphasized their right to withdraw voluntarily at any point. We asked for their informed consent before proceeding with the questionnaire completion. To ensure participant anonymity, we implemented rigorous protocols during the data collection process. For the purposes of this research, any parents, legal guardians, or athletes who did not provide written informed consent were excluded from the onset, and their data were neither collected nor incorporated into the study’s findings. Those who agreed to take part were assigned individual codes to ensure anonymity throughout the data collection period. The questionnaire was administered at three distinct time points: two weeks before the competitive season commenced (T1), at the midpoint of the season (T2), and one week after the end of the competitive season (T3). To ensure unbiased responses, the questionnaires were completed in the absence of their coaches. Participants were encouraged to provide honest answers and seek clarification whenever necessary. Additionally, participants were requested to report their perceptions of their current coach. All data collection procedures strictly adhered to the principles outlined in the Helsinki Declaration. Ethical approval was obtained from the Polytechnic of Leiria Ethics Committee before commencing data collection (Nº CE/IPLEIRIA/24/2021).

### 2.3. Instruments

For the purpose of this study, we utilized the Portuguese version of the Behavioral Regulation Sport Questionnaire [24]. Developed to meticulously assess the self-determined forms of motivation in sports contexts, this questionnaire comprises a total of 24 items. Respondents provide their responses on a seven-point Likert scale, which ranges from 1 (“*Not True for Me at All*”) to 7 (“*Completely True for Me*”). These items are strategically organized into six distinct factors, with each factor housing four items. These factors represent the diverse types of motivation along the motivational continuum. The choice of this specific questionnaire was informed by its proven reliability and validity within sport psychology research. The six subscales—amotivation, external regulation, introjected regulation, identified regulation, integrated regulation, and intrinsic motivation—enable a comprehensive capture of athletes’ motivations. Examples of items include: Amotivation: “*I question why I am putting myself through this*”; External Regulation: “*because people push me to play*”; Introjected Regulation: “*because I would feel guilty if I quit*.”; Identified Regulation: “*because the benefits of sport are important to me*.”; Integrated Regulation: “*because it’s an opportunity to just be who I am.*”; Intrinsic Motivation: “*because I enjoy it*.”

### 2.4. Statistical Analysis

The data analyses were performed using the IBM SPSS Version 26.0 software (IBM Corp., Armonk, NY, USA). Means and standard deviations were reported for continuous variables, while frequency percentages were provided for categorical variables. To assess the deviation from normal distribution, the Kolmogorov–Smirnov test was conducted, and the statistical significance was examined. Internal consistency coefficients (alpha coefficients) were calculated to assess the reliability of the measurement scales used in the study. The acceptable internal consistency was determined with scores above 0.70, indicating a reliable and consistent measurement of the constructs under investigation [25]. A MANOVA repeated measures analysis was performed within the group for all six behavioral regulations. The significance level for rejecting the null hypothesis was set at 5%. To assess the sphericity assumptions, Mauchly’s test was conducted. In cases where this assumption was not met, the Greenhouse–Geisser adjusted values and degrees of freedom were reported, indicated by the presence of decimal degrees of freedom. Following the repeated measures analyses, Bonferroni-adjusted post-hoc tests were performed to examine pairwise comparisons. The effect size, measured using partial eta squared, was calculated, and the assumed reference values were as follows: “small” effect = 0.01, “medium” effect = 0.06, and “large” effect = 0.14. The maximum-expectation test will be utilized to input data whenever possible. Data analysis will be conducted both with and without missing data to ensure a comprehensive examination of the results.

## 3. Results

The total sample size for the study comprised 108 participants (78 male; 30 female). The participants’ mean football experience was 7.08 (SD = 2.46) years. Additionally, their average age was 14.31 years (SD = 1.48). On average, the participants engaged in 3.24 (SD = 0.55) training sessions per week. Additionally, the mean weekly training hours were 288.33 (SD = 54.23), suggesting a considerable variation in the amount of training time invested by the athletes. The frequency distribution of participants across different age categories in the study is as follows: Among the 108 participants, 19 individuals (17.6%) belonged to the “Under 12” category, while 35 participants (32.4%) were classified as “Under 13”. Additionally, there were 19 participants (17.6%) in the “Under 17” group, and the “Under 19” category consisted of 35 participants (32.4%). During the study, data collection from 13 participants at T1 and an additional 8 participants at T2 was hindered due to medical reasons (*n* = 7) and discontinuation from the football club for personal reasons (*n* = 14). Table 1 presents the descriptive statistics and internal consistency coefficient of the behavioral regulations over time. All variables displayed acceptable scores of internal consistency. The variables demonstrated normal distribution, as confirmed with the K–S test (*p* < 0.05).

The results revealed that the sphericity assumption was met for amotivation (W = 0.972, *p* = 0.308), intrinsic motivation (W = 0.977, *p* = 0.384), and external regulation (W = 0.972, *p* = 0.308). However, for introjected regulation (W = 0.795, *p* < 0.001), identified regulation (W = 0.795, *p* < 0.001), and integrated regulation (W = 0.795, *p* < 0.001), the sphericity assumption was violated. To address this, Greenhouse–Geisser-adjusted values and degrees of freedom were reported, ensuring the validity of the statistical inferences in these cases. For introjected regulation, the Greenhouse–Geisser-adjusted value was reported as ε = 0.845. For identified regulation, the Greenhouse–Geisser-adjusted value was ε = 0.845. And for integrated regulation, the Greenhouse–Geisser-adjusted value was also ε = 0.845.

The MANOVA repeated measures analysis showed significant differences in identified regulation and intrinsic motivation between the time points. Specifically, for identified regulation, there was a significant difference between T1 and T3 (*p* < 0.01, η^2p^ = 0.13), indicating an increase in the mean scores over time. Similarly, for intrinsic motivation, there was also a significant difference between T1 and T3 (*p* < 0.01, η^2p^ = 0.12), showing a decrease in the mean scores over time. On the other hand, no significant differences were observed in amotivation, external regulation, introjected regulation, and integrated regulation between the three time points (see Table 2). We conducted the same analysis using the EM algorithm for data imputation to address the missing data. The results obtained from this imputation method were found to be comparable with those reported with missing data, indicating that the EM algorithm adequately handled the missing values. After careful consideration, the research team decided to retain the raw data in the analysis since the sample size was deemed sufficient to perform the necessary statistical procedures.

While not the primary objective of the study, the researchers were intrigued to explore the levels of amotivation among participants who chose to discontinue from football and subsequent assessments. Consequently, three distinct temporal groups were identified: T1 Discontinuation (*n* = 13) for participants who discontinued after the first assessment, T2 Discontinuation (*n* = 8) for those who discontinued after the second assessment, and T3 Full Participation (*n* = 87) for individuals who maintained their football practice and participated in all three assessments. At the first assessment, the T1 Discontinuation group exhibited higher mean amotivation scores (M = 2.36, SD = 0.93) compared to the T2 Discontinuation group (M = 2.06, SD = 1.30) and the T3 Full Participation group (M = 2.20, SD = 1.12). This observation suggests that participants in the T1 Discontinuation group already experienced elevated levels of amotivation early in the study, potentially contributing to their decision to discontinue football practice at this stage. At the second assessment, the T2 Discontinuation group displayed greater mean amotivation scores (M = 2.73, SD = 1.82) compared to the T3 Full Participation group (M = 2.37, SD = 1.34).

## 4. Discussion

The current study aimed to investigate the changes in behavioral regulations and intrinsic motivation over a competitive season (T1, T2, and T3). The results revealed significant differences in identified regulation and intrinsic motivation between these time points. Specifically, identified regulation demonstrated a significant increase in mean scores from T1 to T3, indicating that athletes experienced a higher level of identified regulation over time. On the other hand, intrinsic motivation showed a significant decrease from T1 to T3, indicating a decline in participants’ intrinsic motivation as the study progressed. Throughout the competitive season, all the remaining behavioral regulations, including amotivation, introjected regulation, and integrated regulation, did not show significant changes in these teenage football players.

The results of the study did not provide support for the hypothesis that intrinsic motivation would increase during the competitive season. Contrary to the initial hypothesis, the findings showed a significant decrease in intrinsic motivation between T1 and T3 (*p* < 0.01, η^2p^ = 0.12). As the season progresses and decisive stages approach, the escalating demands may lead to an increased emphasis on extrinsic factors such as results and qualification for subsequent competitions. Consequently, this heightened focus on external contingencies could potentially diminish players’ intrinsic motivation [14,16]. Players may have experienced burnout or fatigue, affecting their intrinsic motivation levels. Additionally, external factors, such as interpersonal behaviors, roles within the team, coaching styles, performance expectations, or overtraining might have influenced the players’ intrinsic motivation [3,6,16]. If players perceived an overly controlling or pressurizing environment, it could have negatively impacted their intrinsic motivation [14].

The outcomes did not provide evidence to support the hypothesis that integrated regulation would experience an increase during the season. No significant differences were observed in integrated regulation across the three time points. One plausible explanation is that the process of integrating football involvement with personal values and identity may require more time than the study’s duration allowed for [9,26]. It is possible that players need additional experiences and exposure to solidify their motivations and align them with their core beliefs and sense of self. Furthermore, external factors like changes in team dynamics or personal life events unrelated to football may have influenced the players’ integrated regulation, leading to fluctuations in motivation and preventing a consistent increase over time [12,16,22].

The findings demonstrated a significant increase in identified regulation between T1 and T3 (*p* < 0.01, η^2p^ = 0.13), supporting the hypothesis that participants’ personal importance and commitment to football involvement strengthened over the competitive season. However, no significant differences were observed between T1 and T2, and T2 and T3. This suggests that identified regulation showed a gradual progression over time, indicating players’ increased engagement with the sport and alignment of their football involvement with their personal values and identity. Various factors, such as enhanced self-determination, may have contributed to this observed increase in motivation levels during the competitive season, emphasizing the need to consider dynamic changes in motivation types over time [4,9,21,22,27].

The results of the study showed no significant differences in introjected regulation between T1, T2, and T3. The initial hypothesis suggested that introjected regulation might undergo changes over the competitive season, potentially increasing initially and then stabilizing or decreasing. However, the data did not provide strong evidence to support this hypothesis, as the differences observed in the introjected regulation scores were not statistically significant (*p* > 0.05). Further research may be needed to explore additional factors that could influence introjected regulation and its fluctuations over time during a competitive season. Introjected regulation is based on internalizing external motivations, often related to feelings of guilt, shame, or self-worth tied to performance. After internalizing these external pressures, they are likely to remain relatively consistent over time, particularly if there are no significant changes in the external context or demands of the sport. This observation is evident from the results related to external regulation and amotivation, which did not show significant variations during the course of the study [16,22].

The findings showed no significant differences in external regulation between the three time points. It is possible that the external factors influencing motivation may have remained relatively stable throughout the season, leading to a lack of significant change in external regulation scores. The impact of external factors on motivation might be influenced by other contextual and situational factors, such as team dynamics, coaching styles, or the overall competitive environment [16,22]. The lack of significant differences in external regulation suggests that the role of external factors in influencing athletes’ motivation during the competitive season may be complex and multifaceted.

The findings indicated no significant differences in the amotivation scores across the competitive season. It is possible that external factors influencing amotivation remained relatively constant throughout the season, preventing a noticeable decline. Additionally, individual differences in players’ initial levels of amotivation and their response to various situational factors may have contributed to the stable pattern of amotivation over time [16]. It is important to recognize that motivation is a complex construct influenced by multiple factors, and a more nuanced investigation might be required to understand the dynamics of amotivation in teenage football players [14,17].

It is important to recognize that amotivation consistently exhibited greater mean scores in comparison to external and introjected regulation, indicating that the participants tended to experience a higher degree of a lack of motivation or disinterest in football throughout the competitive season. During the research, fourteen players withdrew from the study, and their motives could potentially be linked to the scores reported at T1 for amotivation, external regulation, and introjected regulation. Further investigations are necessary to delve deeper into this matter and better understand the potential influence of participant attrition on the observed results.

In our exploration of amotivation, we noticed distinct patterns among participants based on their decisions to continue or leave football. The T1 Discontinuation group had higher initial amotivation scores compared to the T2 Discontinuation and T3 Full Participation groups, leading them to discontinue after the first assessment. The T2 Discontinuation group, while showing heightened amotivation by the second assessment, opted out after T2. On the other hand, the T3 Full Participation group, displaying steady, moderate levels of amotivation, remained committed throughout the season. These patterns highlight the importance of monitoring behavioral motivations among teenage football players over time. By delving deeper into these motivational dynamics, we can gain insights into what propels their decision to stick with or leave the sport [13].

### Limitations and Agenda for Future Research

One notable constraint of the current study is the lack of experimental manipulation or interventions. To address this limitation, future research could incorporate experimental designs aimed at enhancing different types of motivation, such as autonomy-supportive coaching or goal-setting strategies. By implementing such interventions, researchers can then investigate the causal effects of these approaches on the various behavioral regulations observed in teenage football players. Beyond the disparity in the sample sizes of male and female participants, variables such as the participant’s age, playing experience, and psychological skill level (e.g., resilience and coping strategies) can potentially influence their motivation. To address this concern, forthcoming studies might be enriched by employing more homogenous participant groups, particularly by accounting for sex differences, controlling for specific football categories, and recruiting individuals with comparable skill levels or from similar age groups. Furthermore, the data collection process during the competitive season and post-season (relative to the final classification of each team) may have introduced additional variability in the results. This includes conducting an analysis based on the type of competition (regional vs. national). Team performance, success, or failure during the season could potentially influence players’ motivations, leading to varying responses. To gain a more comprehensive understanding of motivational changes, future research should consider collecting data at multiple time points throughout the season to capture the dynamics of motivation in response to fluctuating levels of team success or adversity. Moreover, longitudinal studies could be conducted to assess motivation over an extended period, such as 1–2 years, enabling researchers to gain deeper insights into how motivations evolve throughout an athlete’s career. In our endeavor to quantitatively delineate the behavioral regulations of teenage football players, we recognize the inherent value of qualitative methodological explorations. Consequently, it would be prudent for subsequent research to embrace mixed-method paradigms, integrating both quantitative and qualitative datasets, to yield a more intricate and contextually nuanced understanding of player motivations and experiences.

## 5. Conclusions

The current study aimed to provide valuable insights into the dynamic changes in behavioral regulations among teenage football players over a competitive season. The results highlighted a noteworthy pattern: identified regulation demonstrated a significant increase over time, indicating that athletes’ dedication and personal commitment to the sport intensified as the season progressed. Conversely, intrinsic motivation displayed a significant decrease, suggesting that players’ inherent enjoyment and interest in football declined over the course of the season. Notably, no significant changes were observed in introjected regulation, integrated regulation, and amotivation. These findings offer a valuable understanding of the multifaceted nature of motivation in teenage athletes and emphasize the importance of considering various motivational factors to better comprehend their engagement with the sport.

### Practical Implications

The implications of this study for practical application are twofold. Firstly, coaches and sports practitioners should be attuned to the potential fluctuations in athletes’ intrinsic motivation throughout a competitive season. To counteract declining intrinsic motivation, it is vital to create a supportive and autonomy-promoting environment that fosters a sense of enjoyment and personal fulfillment in football. Secondly, recognizing the increase in identified regulation suggests that athletes may develop a stronger sense of personal significance and dedication to the sport. Coaches can capitalize on this intrinsic commitment by aligning team objectives and training sessions with players’ individual values, thereby enhancing their sense of ownership and dedication to the team’s success. Moreover, the technical co-ordinators of the clubs can schedule dedicated sessions to analyze the data of the various teams and collaborate with different technical staff to devise optimal strategies for each team. Understanding these dynamic changes in motivation can empower coaches and practitioners to tailor their strategies, creating a positive and supportive atmosphere that fosters sustainable motivation and enriches the overall teenage football experience.

## Figures and Tables

**Table 1:** Descriptive statistics and internal consistency coefficient.

Variables		T1			T2			T3	
	M	SD	α	M	SD	α	M	SD	α
Amotivation	2.21	1.12	0.70	2.41	1.30	0.84	2.31	1.34	0.89
External Regulation	1.68	0.91	0.70	1.75	1.07	0.91	1.71	0.88	0.83
Introjected Regulation	1.79	1.12	0.81	2.02	1.23	0.85	1.99	1.19	0.80
Identified Regulation	5.03	1.07	0.71	5.10	1.30	0.82	5.40	1.13	0.80
Integrated Regulation	5.59	1.13	0.79	5.69	1.07	0.83	5.75	1.02	0.87
Intrinsic Motivation	6.41	0.69	0.80	6.32	0.78	0.81	6.16	0.99	0.88

Notes: T = time points; M = mean; SD = standard deviation; α = internal consistency coefficient.

**Table 2:** Mean comparison within measures.

Variables	F	df1	df2	*p*	η^2p^	Pairwise Comparisons
Amotivation	0.64	2	82	0.81	0.01	n.s. (*p* > 0.05)
External Regulation	0.38	2	82	0.63	0.01	n.s. (*p* > 0.05)
Introjected Regulation	1.33	2	82	0.27	0.03	n.s. (*p* > 0.05)
Identified Regulation	5.91	2	82	<0.01	0.13	T1 ≠ T3
Integrated Regulation	0.92	2	82	0.39	0.02	n.s. (*p* > 0.05)
Intrinsic Motivation	5.82	2	82	<0.01	0.12	T1 ≠ T3

Notes: F = F test results; df1 = degrees of freedom; df2 = degrees of freedom; *p* = significance; η^2p^ = partial eta squared; n.s. = no differences detected.

## Data Availability

The data used in this study were obtained under a specific license exclusively for the purposes of this research. While the data supporting the findings of this study are not publicly available, they can be requested and accessed upon reasonable inquiry, subject to permission from the Life Quality Research Center and the corresponding author.
